# 

**Published:** 1896-09

**Authors:** 


*The following donations have been received since the publication of the list in June:*

*August 31st, 1896.*

| | |
|---|---|
| British Medical Association (1) ... ... ... ... | 1 volume. |
| J. Michell Clarke, M.D. (2) ... ... ... ... ... | 1 " |
| George M. Gould, M.D. (3) ... ... ... ... ... | 82 volumes. |
| L. M. Griffiths (4) ... ... ... ... ... ... ... ... | 8 " |
| L'Institut international de Bibliographie (5) ... | 1 volume. |
| C. Moore Jessop (6) ... ... ... ... ... ... ... | 24 volumes. |
| R. Shingleton Smith, M.D. (7) ... ... ... ... ... | 1 volume. |
| P. Watson Williams, M.D. (8) ... ... ... ... | 4 volumes. |

Unbound periodicals have been received from Dr. P. Watson Williams.

### TWENTY-FIRST LIST OF BOOKS.

The titles of books mentioned in previous lists are not repeated.

The figures in brackets refer to the figures after the names of the donors, and show by whom the volumes were presented. The books to which no such figures are attached have either been bought from the Library Fund or received through the *Journal*.

**Allis, O. H.** ... *Dislocations of the Hip* ... ... ... ... ... ... ... ... 1896
**Bahadurji, K. N.** *Medical Administration of India* ... ... ... ... ... ... 1896
**Bannatyne, G. A.** *Rheumatoid Arthritis* ... ... ... ... ... ... ... ... 1896
**Bergey, J. S. Billings, S. W. Mitchell, and D. H.** *The Composition of Expired Air and its Effects upon Animal Life*... 1895
*Bibliographie scientifique, Organisation internationale de la* ... ... ... (5) 1896
**Billings, S. W. Mitchell and D. H. Bergey, J. S.** *The Composition of Expired Air and its Effects upon Animal Life*... 1895
**Broca et P. Maubrac, A.** *Traité de Chirurgie cérébrale* ... ... ... ... 1896
**Brockbank, E. M.** *On Gall-stones* ... ... ... ... ... ... ... ... ... ... 1896
**Cheyne, W. W.** ... *Operations for Cancer* ... ... ... ... ... ... ... ... 1896
**Clarkson, A.** ... *A Text-Book of Histology* ... ... ... ... ... ... .. 1896
**Cooke, T.** ... ... *A Plea for Practical Work in Anatomy* ... ... (4) 1893
**Corfield, W. H.**... *Disease and Defective House Sanitation* ... ... ... 1896
**Dalton, N.**... ... *Aids to Medicine*, Parts I.—IV. ... ... ... 2 vols. [1896]
**Delanglade, É.**... *De la Luxation congénitale du Fémur* ... ... ... ... 1896
**Doyne, R W.** ... *Diseases of the Eye* ... ... ... ... ... ... ... ... ... 1896
**Dunogier, S.** ... *De la Prothèse appliquée au Traitement des Empyèmes de l'Antre d'Highmore* ... ... ... ... .. ... 1896
**Ferguson, [R. S.]** *Official Guide to Carlisle* ... ... . . ... ... (7) 1896
**Fothergill, W. E.** *Manual of Midwifery* ... ... ... ... ... ... ... ... 1896
**Freyer, P. J.** ... *Modern Treatment of Stone* ... ... ... ... 2nd Ed. 1896
**Fripp, H.** ... ... *Retrospect of the Transactions of the Bristol Medico-Chirurgical Society* ... ... ... ... ... (4) [N.D.]
,, ... ... *The Doctrine of Contagium Vivum, in its Relation to Parasitic Disease* ... ... ... ... ... ... (4) 1878
**Gemmell, G. H.**.. *Chemical Notes and Equations* ... ... ... ... ... [1896]
**Goodall and J. W. Washbourn, E. W.** *A Manual of Infectious Diseases* 1896
**Gould, G. M.** ... *Borderland Studies* ... ... ... ... ... ... ... (3) 1896
**Hall, J.** ... ... *Select Observations on English Bodies of Eminent Persons in Desperate Diseases* (Tr. by J. Cook). 3rd Ed. 1683
**Hedley, W. S.** ... *The Hydro-Electric Methods in Medicine* ... 2nd Ed. 1896
**Hewer, Mrs. L.**.. *Our Baby* ... ... ... ... ... ... ... ... ... ... 4th Ed. 1896
**Hyde, S.** ... ... *Rheumatoid Arthritis* ... ... ... ... ... ... ... ... 1896
*Isle of Man, The. Official Guide* ... ... ... ... ... ... ... ... ... ... [N.D.]
[*Jenner, Eduardo*] *Boletin del Consejo superior de Salubridad. Numero especial* 1896
**Jessop, C. M.** ... *Asiatic Cholera* ... ... ... ... ... ... ... (6) 1883
**Johnson, Sir G.**.. *The Pathology of the Contracted Granular Kidney* ... 1896
**Keane, A. H.** ... *Ethnology* ... ... ... ... ... ... ... ... ... ... ... 1896
**Lee, R.** ... ... *On Ovarian and Uterine Diseases* ... ... ... ... (6) 1853
**Lusk, W. T.** ... *The Science and Art of Midwifery* ... ... ... ... (6) 1884
**Macan, A. V.** ... *The Rational Treatment of Anterior and Posterior Displacements of the Uterus* ... ... ... ... (4) 1885
**Maclagan, T. J.**.. *Rheumatism* ... ... ... ... ... ... ... ... 2nd Ed. 1896
**Maubrac, A. Broca et P.** *Traité de Chirurgie cérébrale* ... ... ... ... 1896
*Medical, The Musings of a.* No. I. ... ... ... ... ... ... ... (8) [N.D.]
**Mitchell and D. H Bergey, J. S. Billings, S. W.** *The Composition of Expired Air and its Effects upon Animal Life* ... 1895
**Nall, S.** ... ... *Aids to Obstetrics* ... ... ... ... ... ... 5th Ed. [1896]

*Opium in India, The Consumption of* ... ... ... ... ... ... ... ... ... ... 1895
**Paget, S.** ... ... *The Surgery of the Chest* ... ... ... ... ... ... ... 1896
**Pritchard, U.** ... *Diseases of the Ear* ... ... ... ... ... ... ... 3rd Ed. 1896
**Ramm, F.** ... ... *Kastration ved Prostatahypertrofi* ... ... ... ... ... ... 1896
**Robertson, W. G. A.** *Clinical Diagnosis* ... ... ... ... ... ... ... 1896
**Sandwith, F. M.** *Cholera in Egypt* ... ... ... ... ... ... ... ... (8) 1892
**Smith, R. A.** ... *Air and Rain* ... ... ... ... ... ... ... ... ... (6) 1872
**Smith, W. R.** ... *Angio-Neurosis* ... ... ... ... ... ... ... ... ... ... [N.D.]
*Southport—A Modern City of Health* ... ... ... ... ... ... ... ... ... [N.D.]
**Squire, B.** ... ... *On Lupus Vulgaris* ... ... ... ... ... ... ... ... (4) 1888
**Sturges, O.** ... *Pneumonia* ... ... ... ... ... ... ... ... ... ... (6) 1876
**Swain, P.** ... ... *Surgical Emergencies* ... ... ... ... ... ... 5th Ed. 1896
**Tiebel, H. C.** ... *Official Handbook of Spas, Watering Places, and Health Resorts* ... ... ... ... ... ... ... ... ... [1896]
**Washbourn, E. W. Goodall and J. W.** *A Manual of Infectious Diseases* 1896
**Weber, H. and F. P.** *The Spas and Mineral Waters of Europe* ... ... ... 1896
**Wright, C. D.** ... *Report on the Factory System of the United States* (8) 1884
**Yeo, I. B.** ... ... *Food in Health and Disease* ... ... ... [2nd Ed.] 1896
**Ziemssen, H. v.** ... *Klinische Vorträge* ... ... ... ... ... ... ... (8) 1887-93

---

### TRANSACTIONS, REPORTS, JOURNALS, &c.

Advertisers' Guardian, The ... ... ... ... ... ... ... ... ... ... ... ... ... 1891

American Gynæcological and Obstetrical Journal, The ... Vol. VIII. 1896

American Journal of Obstetrics, The ... ... ... ... Vol. XXXIII. 1896

American Journal of the Medical Sciences, The (3) Vols. VI.—X., 1830-32, XXI.—XXVI., 1837-40, N.S. V.—XII., 1843—46, XV.—XXVIII., 1848-54, XXXI.—LXXVIII., 1856-79; CXI. 1896

Année psychologique, L' ... ... ... ... ... ... ... ... ... ... ... (2) 1895

Archives de Neurologie ... ... ... ... ... ... ... ... 2e Sér. Tome I. 1896

Asclepiad, The ... ... ... ... ... ... ... ... ... ... ... ... (4) Vol. V. 1888

Australasian Medical Gazette, The ... ... ... ... ... ... Vol. XIV. 1895

Birmingham Medical Review, The ... ... ... ... ... Vol. XXXIX. 1896

Boston Medical and Surgical Journal, The ... ... Vol. CXXXIV. 1896

Bristol Health Report ... ... ... ... ... ... ... ... ... ... (4) 1891; 1896

British Gynæcological Journal, The ... ... (1) Vols. I., 1886; XI. 1895

British Medical Journal, The ... ... ... ... ... ... ... ... Vol. I. for 1896

Buffalo Medical Journal ... ... ... ... ... ... ... ... ... Vol. XXXV. 1896

China Medical Missionary Journal, The ... ... Vols. VIII., IX. 1894-95

,, ,, ,, Indices to Vols. I.—IX., 1887-95 1896

Cincinnati Lancet-Clinic, The ... ... ... ... ... ... ... Vol. LXXV. 1896

Dictionary of Bathing Places, Bradshaw's ... ... ... ... ... ... ... ... 1896

Dictionary of the World's Press, Sell's ... ... ... ... ... ... ... ... 1892

Dublin Journal of Medical Science, The ... ... ... ... ... Vol. CI. 1896

Edinburgh Medical Journal ... ... ... ... ... ... ... ... Vol. XLI. 1896

Grant College Medical Society, Bombay, Transactions of the ... ... 1896

Hospital and Charities Annual, Burdett's ... ... ... ... ... ... ... ... 1896

| | | |
|---|---|---|
| Hospitals— | | |
| Cincinnati Hospital, Thirty-fifth Annual Report of the | | 1896 |
| Edinburgh Hospital Reports | Vol. IV. | 1896 |
| Journal of Laryngology, Rhinology, and Otology, The | Vol. X. | 1896 |
| Journal of the American Medical Association, The | Vol XXVI. | 1896 |
| Lancet, The | Vol. I. for | 1896 |
| Medical and Surgical Reporter, The | Vol. LXXIV. | 1896 |
| Medical Chronicle, The | N.S. Vol. IV. | 1896 |
| Medical Press and Circular, The | [Vol. CXII.] | 1896 |
| Medical Record | Vol. XLIX. | 1896 |
| Medical Register, The | | 1883 |
| Montreal Medical Journal, The | [Vol. XXIV.] | 1896 |
| Newspaper Press Directory | | 1889 |
| New York Medical Journal, The | Vol. LXIII. | 1896 |
| New York, Transactions of the Medical Society of the State of | | 1896 |
| Pediatrics | Vol. I. | 1896 |
| Progrès médical, Le | 3e Sér. Tome III. | 1896 |
| Retrospect of Medicine, Braithwaite's | Vol. CXIII. | 1896 |
| Revue obstétricale et gynécologique | | 1896 |
| St. Louis Medical and Surgical Journal, The | Vol. LXX. | 1896 |
| Sanitary Record, The | Vol. XVII. | 1896 |

### Local Medical Notes.

### BRISTOL.

Health Report.—The annual Report of the Medical Officer of Health for 1895 has been recently distributed, and contains much valuable and interesting information on the health of the city and the means which are taken to keep the condition of the health-rate up to its present highly satisfactory state.

Part I. is the "Report of the General Sanitary Condition of the Urban Sanitary District of Bristol." We are glad to find that the *old wells* which still exist are gradually being closed. All of those examined (43) during the year were found to be more or less polluted—a fact we can hardly be surprised at when we remember that the soil has been lived over for centuries. In one case the users had attempted to purify the water by a charcoal filter, with the result of making it fouler than before.

As many of our readers know, the loan (£16,100) for the completion of the *Smallpox Hospital* upon the Novers Hill site (13 acres) has received the sanction of the Local Government Board. This will add 20 beds to the already existing 50, as well as administration, isolation and laundry blocks, and a disinfecting station.

The inquiry into the Ham Green site (20½ acres) was progressing at the time the report was written, the proposed loan being £27,000. The permission has since been granted and the work was begun at once. This will add 76 beds to the present accommodation.

The calculated population of Bristol is now 228,139. During 1895 there was an increase in the *birth-rate*, which had previously been falling off—6,622 births, 29.02 birth-rate, compared with 6,393 births, 28.81 birth-rate in 1894; while the *marriage-rate* has fallen off, 10.8 against 11.5 in 1894. *Illegitimate births*, however, have risen to 222 from 189. The number of *deaths* has risen to 4,108 from 3,888, or 18.08 as compared with 17.16—the lowest ever recorded—in 1894. *Infant mortality* has fallen from 948 to 935; the rate is therefore lower—141.19 against 148.31 in 1894. Bristol as usual compared favourably with many big towns. According to the averages, which at best are most deceptive things, 302 persons now living should be dead. We congratulate these individuals, whoever they may be. This net gain is lower than 1894, when it was 550.

As is usually the case, the latest available returns of *vaccination* are those of the previous year (1894), and, as may be easily explained, a considerable falling off is observed, the percentage successfully vaccinated being 71.49 as compared with 76.91. The figures are for the Bristol Union. For the Barton Regis Union the numbers are still lower, 68.6 against 72.0 in 1893.

Owing to the hot summer and autumn, there was a great increase in fatal cases of *diarrhœa*, this being especially marked in the last two quarters of the year, 143 cases in 1895, as compared with 65 in 1894.

*Diphtheria* cases increased slightly, 165 against 129 in 1894 and 142 in 1893; but the mortality appears to have decreased, 20.6 against 39 in 1894.

The bacteriological examination of the cases by Dr. Dowson is interesting. He examined 87 cases, finding the bacillus in 70, and as in the remaining 17 no opportunity was given for a second examination, it is possible they may have been true cases of diphtheria. The medical Officer of Health calls attention to the fact that the examination is to confirm, not to make a diagnosis. We commend to the opponents of enlarging the city boundaries Dr. Dowson's remarks on the incidence rate of diphtheria in the various Bristol districts, especially the following:—"Considering the amount of infection that comes into Bristol from surrounding districts, the present system is rather one of keeping disease within certain limits than of preventing its occurrence."

Only two cases of *smallpox* occurred in the city, but two were received from outside authorities for isolation. We are promised a special report later.

*Erysipelas*, the notification of which the Medical Officer thinks unnecessary, shows a slight increase, as does *scarlet fever*, though there was no epidemic of the latter disease. No case of *typhus* has been known to exist in the city since the 1889-90 winter. The falling off in the number of *typhoid* cases is satisfactory, due no doubt to the better sanitary conditions in general. Dr. Davies does not think that with our present hospital accommodation the time is ripe for the notification of *measles*. Only 8 deaths occurred from this disease in 1895.

There is a slight falling off in the number of deaths from *phthisis*, but the number is still large—in fact more than those caused by the seven principal zymotic diseases.

In comparing Bristol with other large towns our city comes out very satisfactorily; but we feel we have already overburdened this notice with figures, and must refer to the comparative tables and comments thereon for further information.

In Part II. is the Report of the Inspector of *Nuisances*, and we can only speak in unqualified praise of the way in which this department has been worked. We believe that the public are beginning to recognise that it is to their own advantage to follow the requirements of the various Acts which insist upon their premises being kept in a condition conducive to health, and consequently the work of the department, though it may increase in amount, is rendered easier by the more ready compliance of the owners of workshops and other premises.

The whole Report shows how constantly and zealously our Public Health Department works for the public good. Mr. Rintoul, of Clifton College, as usual gives us his *meteorological observations*. The chief peculiarities of 1895 were the very cold February—the mean temperature of which was 11.51° below the normal—and the extraordinary warm September, the highest temperature of the year being 82.2° on the 24th. The rainfall was slightly below the average.

University College.—The following students have passed examinations:—

**University of London.**—*Preliminary Scientific* (*M.B.*) *Examination. Entire Examination*—(Second Division), Herbert James Slade, John Elliott Sparks. *Intermediate Examination. Entire Examination*—(First Division), Miles Harris Phillips. (Second Division), William Barrington Prowse; *Excluding Physiology*—(First Division), Thomas Aubrey; *Physiology only*—(Second Division), Arthur Bernard Cridland.

**Examining Board in England by the Royal Colleges of Physicians and Surgeons.**—*First Examination. Part I. Chemistry and Physics*—Reginald Reynalds, James Christopher Wadmore; *Part III. Elementary Biology*—Reginald Reynalds. *Second Examination. Anatomy and Physiology*—Charles Herbert Welch.

**Army Medical Service.**—At the recent examination Mr. G. E. F. Stammers was placed first on the list.

**Indian Medical Service.**—At Netley in July Mr. A. E. H. Pinch's combined marks secured him the third place in the examination. He gained the Herbert Prize of £20, the Prize in Pathology presented by Surgeon-General Sir Joseph Fayrer, Bart., K.C.S.I., the de Chaumont Prize in Hygiene, and the 2nd Montefiore Prize.

**Obituary.**—It is with much regret we have to chronicle the sad news that cholera has carried off two prominent Bristol School men since our last issue, both of them well known and respected in the services on account of their medical successes, and both of them remarkable for the keen interest they took in all kinds of sport.

Surgeon-Major Cecil Henderson of the Indian Medical Service, fourth son of the late Alfred Henderson of Stoke Bishop, died at Sangor, Central Provinces, India, on July 16th, in his forty-second year, leaving a widow and two children.

Surgeon-Captain John Ernest Trask of the Army Medical Staff, after serving in India for five years (where he was captain of the Poona Gymkhana cricket eleven), took up an appointment in 1895 in the Egyptian Army. During the late outbreak of cholera in the Soudan he was indefatigable in his attention to the sick; he contracted the disease and died at the early age of thirty-four.

### *PUBLICATIONS RECEIVED.*

From BAILLIÈRE, TINDALL & COX, London:
*Aids to Medicine.* Parts I.—II. and III.—IV. By NORMAN DALTON, M.D. [1896.]
*Modern Treatment of Stone.* By P. J. FREYER, M.A., M.D., M.Ch. Second Edition. 1896.
*Chemical Notes and Equations.* By G. H. GEMMELL. [1896.]
*Operations for Cancer.* By W. WATSON CHEYNE, M.B., F.R.S. 1896.
*Aids to Obstetrics.* By SAMUEL NALL, B.A., M.B. Fifth Edition. [1896.]

From JOHN BALE & SONS, London:
*Rheumatoid Arthritis.* By SAMUEL HYDE, M.D. 1896.

From BENNETT BROTHERS, LD., Bristol:
1895. *City and County of Bristol. Annual Report of the Medical Officer of Health.* 1896.

From ADAM AND CHARLES BLACK, London:
*Rheumatism.* By T. J. MACLAGAN, M.D. Second Edition. 1896.

From A. BONNER, London:
*The Anomalies and Evils of the Medical Administration of India.* By K. N. BAHADURJI, M.D. 1896.

From BROWN & SONS, LIMITED, Douglas:
*The Isle of Man Official Guide.* [N.D.]

From BURGOYNE, BURBIDGES & CO., London:
*Creosotal (Crecsote Carbonate) and Guaiacol Carbonate.* [N.D.]
*Xeroform.* By Dr. MED. E. HEUSS. [N.D.]

From CASSELL AND COMPANY, LIMITED, London:
*Food in Health and Disease.* By I. BURNEY YEO, M.D. [Second Edition.] 1896.

From J. & A. CHURCHILL, London:
*Surgical Emergencies.* By PAUL SWAIN. Fifth Edition. 1896.
*On Gall-stones.* By EDWARD MANSFIELD BROCKBANK, M.D. 1896.
*Aural Surgery.* By Sir WILLIAM B. DALBY, M.B. Third Edition. 1896.

From CHURCH MISSIONARY HOUSE, London:
*Medical Mission Quarterly.* No. XV. July, 1896.

From WILLIAM F. CLAY, Edinburgh:
*Manual of Midwifery.* By W. E. FOTHERGILL, M.A., B.Sc., M.B., C.M. 1896.

From THE COMMERCIAL GAZETTE JOB PRINT, Cincinnati:
*Thirty-Fifth Annual Report of the Cincinnati Hospital.* 1896.

From CORNISH BROTHERS, Birmingham:
*The Enlarged Cirrhotic Liver.* By ARTHUR FOXWELL, M.A., M.D. [1896.]

From "EL CORREO ESPAÑOL," Buenos Ayres:
*Anales del Departmento nacional de Higiene.* Junio 24, Julio 1°, 8, 16, 24, Agosto 1° de 1896.

From THE CYCLE PRESS, LTD., London:
*The Cycle.* Vol. V., No. 135. July 11, 1896.

From W. J. DORNAN, Philadelphia:
*An Inquiry into the Difficulties Encountered in the Reduction of Dislocations of the Hip.* By OSCAR H. ALLIS, M.D. 1896.

From O. H. DREYER, St. Louis:
*Medical Review.* June 6, 13, 20, 27, July 4, 11, 18, 25, August 1, 8, 15, 22, 29, 1896.

From EDUARDO DUBLAN, Mexico:
*Boletin del Consejo superior de Salubridad.* Diciembre 31 de 1895; Enero 31, Febrero 29, Marzo 31, Abril 30, Julio 31 de 1896.

From EMMISON BROS., Manchester:
*A Modern City of Health. Southport.* [N.D.]

From IGNACIO ESCALANTE, Mexico:
*Boletin del Consejo superior de Salubridad.* Mayo 22 de 1896. Numero Especial.

From 1422 FOLSOM STREET, San Francisco:
*California Medical Journal* July, August, 1896.

From Dr. L. WEBSTER FOX, Philadelphia:
*Intra-ocular Growths.* By L. WEBSTER FOX, M.D. 1896. [Reprint.]
*Operations Performed in the Eye Department of the Medico-Chirurgical Hospital.* By L. WEBSTER FOX, M.D [N.D.] [Reprint.]
*Valedictory Address to the Graduating Class of the Medico-Chirurgical College of Philadelphia.* By L. WEBSTER FOX, M.D. 1896. [Reprint.]

From G. GONNOUILHOU, Bordeaux:
*De la Prothèse appliquée au Traitement des Empyèmes de l'Antre d'Highmore.* Par le Dr. SIMON DUNOGIER. 1896.

From "ILLUSTRATED LONDON NEWS" OFFICE, London:
*The English Illustrated Magazine.* July—September, 1896.

From "THE INDIAN MEDICAL RECORD," Calcutta:
*The Consumption of Opium in India.* 1895.

From JEYES' SANITARY COMPOUNDS CO., LTD., London:
*Branalcane.* June, 1896.

From KNOLL & CO., Ludwigshafen:
*Ueber den klinischen Werth des Tannalbin.* Von Prof. Dr. O. VIERORDT. 1896. [Reprint.]

From "THE LANCET" OFFICE, London:
*Difficulties under the Infectious Disease (Notification) Act.* 1895.
*Report of "The Lancet" Special Commission on the Relative Strengths of Diphtheria Antitoxin Serums.* 1896.
*Report of "The Lancet" Special Commission on the Relative Efficiency and Cost of Plumbers' Work.* 1896.
*The Battle of the Clubs. By the Special Commissioner of "The Lancet."* [1895—96.]
*"The Lancet" and the Hyderabad Commissions on Chloroform.* [N.D.]

From CHARLES LETTS & CO., London:
*Dr. Tiebel's Official Handbook of Spas, Watering Places, and Health Resorts.* [1896.]

From H. K. LEWIS, London:
*Diseases of the Ear.* By URBAN PRITCHARD, M.D. Third Edition. 1896.
*Disease and Defective House Sanitation.* By W. H. CORFIELD, M.A., M.D. 1896.
*Diseases of the Eye.* By ROBERT W. DOYNE. 1896.
*A Manual of Infectious Diseases.* By E. W. GOODALL, M.D., and J. W. WASHBOURN, M.D. 1896.

From MORTON & BURT, London:
*Sixty-First Annual Report of the British Medical Benevolent Fund, for the Year* 1895.

From OLIVER AND BOYD, Edinburgh:
*Cliniques.* By ALEXANDER JAMES, M.D. 1896.

From PARKE, DAVIS & CO., Detroit:
1895—6. *Complete Catalogue of the Products of the Laboratories of Parke, Davis & Co.*

From YOUNG J. PENTLAND, Edinburgh:
*Edinburgh Hospital Reports.* Vol. IV. 1896.

From THE REBMAN PUBLISHING COMPANY, LIMITED, London:
*Archives of Clinical Skiagraphy.* No. 2—Vol. I. June, 1896.

From JOHN MORGAN RICHARDS, London:
*Medical Reprints.* June—August, 1896.

From Dr. HUNTER ROBB, Cleveland:
*A Brief Description of the new Lakeside Hospital.* 1896. [Reprint.]

From AUG. SIEGLE, London:
*The Therapist.* Vol. VI., No. 7. 15th July, 1896.

From THE SMITHSONIAN INSTITUTION, Washington:
*The Composition of Expired Air and its Effects upon Animal Life.* By J. S. BILLINGS, M.D., S. WEIR MITCHELL, M.D., and D. H. BERGEY, M.D. 1895.

From G. STEINHEIL, Paris:
*De la Luxation congénitale du Fémur.* Par le Dr. ÉDOUARD DELANGLADE. 1896.

From THE "TATVA-VIVECHAKA"-PRESS, Bombay:
*Transactions of the Grant College Medical Society, Bombay, from January to December* 1895.

From JOHN WRIGHT & CO., Bristol:
*The Surgery of the Chest.* By STEPHEN PAGET, M.A. 1896.
*Angio-Neurosis.* By W. RAMSAY SMITH, M.B., C.M., B.Sc. [N.D.]
*Rheumatoid Arthritis.* By GILBERT A. BANNATYNE, M.D. 1896.
*Our Baby.* By Mrs. LANGTON HEWER. Fourth Edition. 1896.

